# Long-Term Attitude Change After a Single-Day Manager Training Addressing Mental Health at the Workplace

**DOI:** 10.3390/ijerph16245105

**Published:** 2019-12-13

**Authors:** Elena Schwarz, Birgitta Schiller, Kathrin Moertl, Katja Weimer, Marlene Eisele, Johanna Kauderer, Falko Papenfuss, Harald Guendel, Michael Hoelzer

**Affiliations:** 1 Department of Psychosomatic Medicine and Psychotherapy, University Medical Center Ulm, 89081 Ulm, Germany; katja.weimer@uni-ulm.de (K.W.); harald.guendel@uniklinik-ulm.de (H.G.); 2 Department of Psychotherapy Science, Sigmund Freud University Vienna, 1020 Vienna, Austria; birgitta.schiller@sfu.ac.at (B.S.); kathrin.moertl@sfu.ac.at (K.M.); 3 Robert Bosch GmbH, 70839 Gerlingen, Germany; marlene.eisele@de.bosch.com (M.E.); johanna.kauderer@de.bosch.com (J.K.); falko.papenfuss@de.bosch.com (F.P.); 4 Sonnenberg Klinik gGmbH, 70597 Stuttgart, Germany; hoelzer.michael@sonnenbergklinik.de

**Keywords:** common mental disorder, workplace intervention, SMI, social distance

## Abstract

Mental health problems have become one of the most common causes of incapacity for work, and engender high costs to society. Especially managerial behavior was found to have a great impact on employees’ well-being. In order to support those in leading positions in dealing with their own, as well as their employees’, psychological stress factors, we conducted a specific manager training. At the same time, we wanted to find out about the training’s short- and long-term effects. Participants were asked to give information about their knowledge and attitudes concerning mental health (Mental Health Knowledge Schedule, Social Distance Scale), as well as to comment on their own health condition (12-Item Short Form Health Survey, Patient Health Questionnaire) and working situation (Effort–Reward Inventory, Irritation Scale). Data were collected at baseline, as well as 3 and 12 months after the training. Results show long-term improvements in knowledge and attitudes measured by the Mental Health Knowledge Schedule (MAKS: *M_t1_* = 22.88, *Mt2* = 23.79, *Mt3* = 23.79, *p* = 0.005) but not in the Social Distance Scale (SoDi: *M_t1_* = 0.96, *Mt2* = 0.85, *Mt3* = 0.84, *p* = 0.165). Over the period of time observed, no changes were found regarding health- or work-related instruments. Due to the uncontrolled design of the study, further research is needed to determine the exact effectiveness.

## 1. Introduction

This article illustrates the results of a 1-year survey investigating a workplace intervention for managers. Preliminary analyses concerning the managers’ own mental health have already been published in the course of the data collecting process [1].

Mental disorders have been discussed as being one of the main reasons for causing high rates of absenteeism, disability benefits, and early retirement [2]. In 2018, mental health problems caused about 15% of sick leave days in Germany [3]. Thus, they are still one of the leading causes of incapacity for work, and come along with high expenses for the healthcare system, as well as for the employers. Despite this, a high proportion of people with mental health problems do not receive adequate treatment. Attitudinal barriers and a lack of knowledge hinder patients from taking advantage of the offers provided by the healthcare system [4,5]. For employees, concerns about confidentiality and negative impacts on their career may enhance this effect [6].

Meanwhile, mental health at the workplace has gained more and more public and political interest [7]. Investigations have shown the clear impact of working conditions on workers’ health [8]. In the last decade, several studies were performed to identify work-related risk factors causing common mental health disorders, such as depression, anxiety, and stress-related disorder [9]. On the one hand, work requirements such as high job strain, high job demands, long working hours, and low job control were shown to have a significant impact on the development of stress-related and depressive symptoms [10,11,12]. Effort–reward imbalance, role conflicts, low workplace justice, or job insecurity also predicted the occurrence of stress-related disorders [10,11,12,13,14]. Social factors such as conflicts, bullying, and low co-worker or supervisor support showed significant positive associations with stress, anxiety, and depression [10,11,12,15,16]. On the other hand, there is also evidence for mental health benefits of employment [17]. Compared to working citizens, those unemployed have a lower psychological well-being [18] and a greater risk of morbidity [19]. When finding employment, people with mental ill-health report to feel better in the first place [20]. They benefit from a sense of purpose and a greater autonomy, acceptance, and status within society. In addition, positive social interactions at work make them feel welcome, respected, and supported. As far as healthy people are concerned, especially supervisory behavior may result in satisfaction and well-being [21,22]. Supervisory fields of investigation were work support, interpersonal interaction, as well as social and emotional support. Moreover, spending most of the day at work allows colleagues or supervisors to recognize first stress symptoms and behavioral changes at an early stage, and thus be treated rapidly. Comparable to physical diseases, early intervention is proven to be one of the most important factors in the treatment of mental disorders [23].

Trying to address workplace-related risk factors for common mental disorders, a wide range of strategies have been evolved and applied in recent years [24,25,26,27]. The necessity of bringing the topic into the companies was not least underlined by adding an obligatory hazard assessment for mental health risks at the workplace by means of an amendment of the German Working Conditions Act in 2013. Several investigations have addressed the efficacy of the attempted approaches [24,25,26,27]. Thus, primary prevention interventions, such as enhancing the employee’s control or promoting physical activity, have been found moderately effective [25]. Stronger evidence was found for stress management interventions (SMI) based on cognitive behavioral therapy (CBT) elements. With medium-to-large effect sizes, SMIs have shown positive influence on participants’ mental health parameters [28,29]. Also, investigations concerning stigma reduction programs have been found to be effective in improving workplace attitudes [30,31].

As mentioned above, the supervisor in charge may play another important role. Extensive research has been performed on the influence of supervisor behavior and leadership style on employees’ work performance and well-being [32,33,34]. Data show that both can be related to workers’ mental health in a negative or positive way. Also, investigations revealed an influence of leadership behavior on stigma and practical barriers when it comes to mental health treatment [35]. Considering these findings, the inclusion of supervisors is thus crucial in workplace mental health management. It not only needs the support, but also the active participation of the company’s managers. In the last years, specific trainings for those who are responsible for other employees came to the fore [36]. For instance, Angerer and colleagues investigated an SMI for managers in a company in Germany [37]. It was modified from a manualized SMI for larger companies by Siegrist and Silberhorn [38] and mainly focused on three different aims: Improving the awareness of own physical tension, analyzing stress-provoking situations using psychodynamic and CBT techniques, and teaching of established self-management techniques. A decreased level of perceived stress was still found 1 year after the training.

We assumed that managers who supervise their teams on a regular basis should not only know about their own stress management, but also be able to detect significant changes in their employees’ behavior. Furthermore, they should know about specific symptoms that indicate mental impairments. Finally, managers should feel comfortable and confident enough to seek talk with the employees, if necessary. Therefore, a one-day training for managers was implemented within a large company in Germany. Within this naturalistic pilot study, we aimed to examine the effects of the manager training. We expected to find sustainable improvements in the managers’ knowledge and attitudes concerning mental health. Therefore, we chose an observation period of 12 months. As we also focused on the managers’ own stress management for a short time of the training, we additionally wanted to investigate possible positive effects on the participants’ working situation.

## 2. Materials and Methods 

The methods, as well as some of the material, have already been published in a previous article [1]. Thus, the following section refers to the procedure described summarizing and complementing the main facts.

### 2.1. Sample

**Figure 1:**
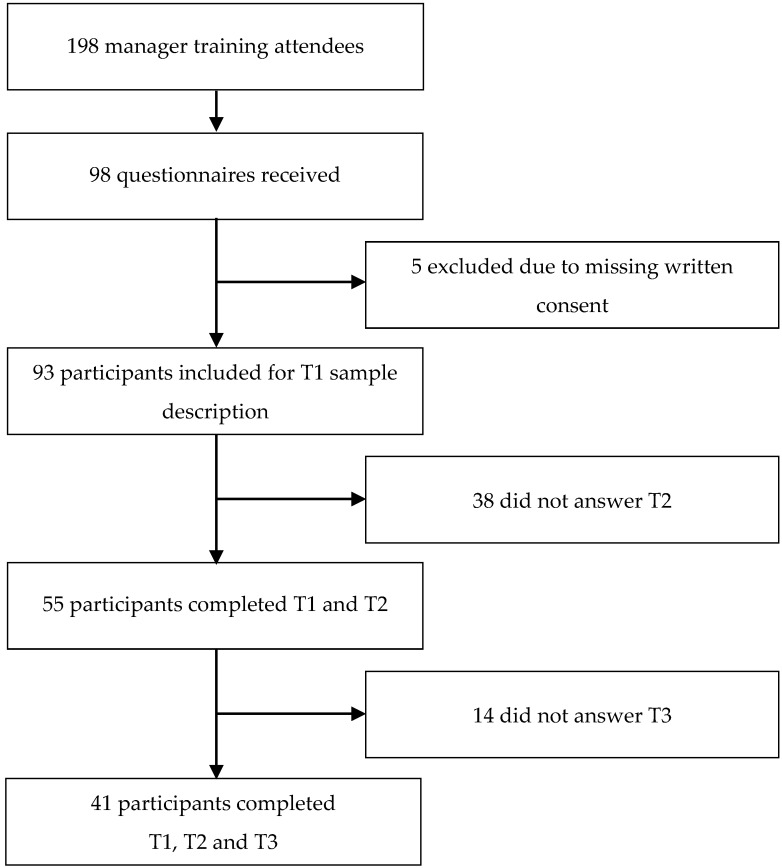
Selection process dataset. T1–T3 measurement points at baseline, and 3 and 12 months after the training.

The sample examined in this study was taken out of a large group of managers who participated in a specific manager training at their company from October 2016 until August 2018. The training was provided at three locations of the industrial company housing headquarters, research and development, as well as purchasing and logistics. In advance, people were invited by email to participate in the study and again asked to take part at the beginning of the training. Out of *N* = 198 training participants in total, *n* = 98 (49.5%) sent back the completed questionnaires, whereas five did not give written consent to the data processing and thus had to be excluded. Data were collected at three points in time: Before the intervention (T1), and 3 (T2) and 12 months (T3) after the intervention. See Figure 1 for an overview. The positions of the managers ranged from team leader (the lowest) to head of department (second highest) within the company.

### 2.2. Manager Training

As part of a company agreement concerning psychosomatic health at the workplace, the one-day workshop was provided and organized by the company’s medical and social services. Each group allowed for 15 managers at maximum. The focus of the intervention was not only the participants’ mental health. but also their handling of difficult situations with employees that might suffer from a mental health problem. The training day mainly comprised three parts aiming at the following goals: (1) To provide sufficient information about the company agreement and internal support structures. (2) To enhance the managers’ awareness of their own, as well as their employees’, mental health. (3) To support the managers in handling difficult situations with their employees. As the training also included group discussions about personal experiences of the managers, all participants were asked to verbally give a confidentiality agreement.

The first part of the program was held by a company staff member. This block served to explain the legal framework and how to put the stipulations of the agreement into practice. Also, managers were told about the structural changes that were introduced within the company agreement. For example, the agreement specifies a step-by-step approach to support the handling of difficult situations and developments. In the mean-time, participants got to know relevant contact persons on site that were educated to support this approach (inhouse doctor, social services, members of the workers’ council, etc.). The second and third part of the training day was performed by two external clinical experts (H.G., M.H.).

In the second part, the expert started discussing topics about mental illness, putting them into workplace context, mainly focusing on the perception and possible consequences of stress, as well as depression and burnout syndrome. Here, various didactic techniques, e.g., initial psychoeducation and interactive lectures, were applied. A self-awareness exercise about the managers’ own early stress symptoms followed. Afterwards, a lively group discussion focused on self-chosen exemplary situations of more chronic stress. Based on cognitive behavioral therapy (CBT) strategies for situation analysis, the managers were then taught to better identify their own thoughts, feelings, and, especially, their bodily reactions. Furthermore, different possibilities to build up resilience were discussed among participants (“strengthening one’s own health”).

Part three of the training was conducted in the afternoon. Again, the focus was set on real experiences of the participants. Managers were asked to talk about current difficult situations concerning their employees with regard to chronic stress-related symptoms or first symptoms of mental illness. Cases were discussed in depth within the group. During the discussion, the expert added professional information about how to deal with employees displaying initial behavioral problems (“paying attention to health of employees”). The expert focused, in particular, on improving the managers’ communication skills. This part was oriented towards a more psychotherapeutic approach (e.g., active listening, structuring a conversation, or how to deal with emotions; role play).

At the end of the training day, a short information block served to give further information on the company’s help offers and was held by one of the industrial council members.

### 2.3. Quantitative Instruments

The questionnaire consisted of two parts. The first part reflects the participants’ knowledge and attitudes concerning mental health. The second part deals with the state of their own physical and mental health, as well as the current working situation. For this purpose, a combination of several validated instruments was selected and complemented by various single item questions. Additionally, socio-demographic data and contact details were collected at baseline.

Knowledge and attitude concerning mental health were measured by the Mental Health Knowledge Schedule (MAKS, [39]) and the Social Distance Scale (SoDi, [40]). MAKS is a short instrument that addresses stigma-related knowledge about mental health using six items (e.g., “Medication can be an effective treatment for people with mental health problems”) [39]. Item scores are summed up to a global score (range 6–30), whereby higher scores represent a better knowledge. MAKS is described as a valid instrument to measure stigma-related knowledge and prejudice. Authors suggest to use the scale in conjunction with attitude measures to learn more about the interaction of knowledge and attitudes. SoDi is a compact tool, firstly developed by Bogardus to measure individual attitudes concerning another social group [41]. Focusing on the general willingness to socially interact with this specific group, SoDi has been validated and used in multiple composures and surveys. Thereby, the desire to avoid contact with members of this group is interpreted as a measure of stigmatizing [42]. Here, we used the 5-item version introduced by Link and colleagues [40], addressing mentally ill people as the interaction group (e.g., “How willing would you be to make friends with the person?”). The SoDi score is the mean value of the items (range 0–4), whereby lower scores mean a higher willingness to interact. In addition to these two validated instruments, we added some further single questions addressing the participants’ attitudes concerning their own mental health and the perceived interaction with mentally ill people in their company (STIGMA, see Appendix A). The items were used in previous research projects in our department. Answers were given on 9-point Likert scales and interpreted separately for every single item (e.g., “Colleagues with mental health problems are supported by colleagues and supervisors and treated fairly”).

To observe the physical and mental health condition of the participants, the 12-Item Short Form Health Survey (SF-12, [43]) and the Depression Scale of the Patient Health Questionnaire (PHQ-D, [44]) were used. The SF-12 addresses the participant’s subjective perception of his or her daily level of functioning (e.g., “In the past four weeks, did you experience any difficulties at work or during any other daily activity according to your physical health?”) [43]. Two main scores of physical and mental health can be calculated by summing up the item scores (range 0–100). Lower scores represent better levels of functioning. The PHQ-D enquires about the occurrence of depressive symptoms during the preceding two weeks (e.g., “fatigue and the feeling of loss in energy”) [44]. The scores of the nine items are summed up with a range of 0–40. According to the diagnostic criteria of the DSM-IV, a cut-off score of 10 indicates a need for treatment [45].

The participants’ current working situation was measured by the German version of the Effort–Reward Inventory (ERI, [46]) and the Irritation Scale (IS, [47]). The ERI is a psychometrically well-justified instrument [48] that enquires psychosocial working strains through two different scales: The effort, that needs to be invested in the daily work (range 6–30), and the possible reward, that comes along with the work (range 11–55) [46]. Sum scores can be analyzed separately or set into relation. Also, the tendency to consider work too much can be measured on a third scale (overcommitment, range 4–24). IS measures the emotional and cognitive strain that participants perceive related to their employment (e.g., “Even at home I think about working issues”) [47]. Various investigations prove IS to be a reliable and valid instrument. The global score is the mean score of the single items (range 1–7).

At the end of the questionnaire, participants were offered to get individual feedback on their personal health condition (SF-12, PHQ-D) and their current working situation (ERI, IS). Feedback was given by comparing the individual scores with sex- and age=specific norm samples and classifying them into five categories according to standard deviation (SD): More than one (two) SD lower than the average, in between the average, and more than one (two) SD higher than the average.

### 2.4. Qualitative Instruments

In addition to the quantitative instruments, semi-structured interviews were conducted with 20 of the participants included in the quantitative survey that will not be reported within the present article. Addressing the implementation process of the manager training, interviews were conducted at baseline and 12-month follow-up, and took about 30 min each. Participants were asked about their motivation for taking part in the training and whether they think the training would pay off for their company. Also, they were invited to talk about their personal way of dealing with employees who suffer from mental health problems. A detailed analysis of the qualitative data will be reported in another place.

### 2.5. Implementation

The intervention formed part of the company’s general training program for managers. Therefore, information was provided on the company’s intranet. Managers voluntarily signed in for the single-day workshop. All trainings took place in seminar rooms located at the company’s site. Rooms were equipped with both a computer and a projector, and offered sufficient space for the group. A few days in advance, participants were informed about the associated study. They received an email already including the questionnaires. Again, on the day of the workshop, the trainer introduced the study and handed out detailed information, a consent form, the questionnaire in printed form, and a prepaid envelope. At the beginning of the training, the managers were given 10 min to either fill in the first half of the questionnaire or, in case they disagreed to the participation, to use the delay as they liked. Due to lack of time, participants were told to answer the second half of the questions later and send it back to the researchers’ department by using the already addressed and prepaid envelopes. For the following data selection procedure, participants were pseudonymized. Only one researcher of the researching team knew about the assignment of the codes to the participants’ contact details. Three and 12 months after the training, all study participants received another package of documents by mail, including the follow-up questionnaire and a prepaid envelope. If requested, participants received feedback regarding their health-related questionnaires via mail.

### 2.6. Design and Data Analysis

The present study was performed in a naturalistic design. For the description of the sample, only baseline information was considered. Any changes of the target variables (knowledge/attitude concerning mental health and perceived working conditions) were analyzed using general linear models for repeated measure designs with an alpha set to 0.05 and Bonferroni-adjusted post-hoc tests to account for multiple testing. All data management and statistical analyses were conducted using IBM SPSS statistics 24 (IBM Corporation, Armonk, NY, USA).

### 2.7. Ethical Approval and Registration

The study was conducted in accordance with the Declaration of Helsinki and the study protocol was approved by the Ethics Committee of Ulm University (326/16). The investigation was registered by the German Clinical Trial Register (DRKS) under ID number DRKS00011371.

## 3. Results

### 3.1. Descriptives

Socio-demographic information at baseline is presented in Table 1. An overview of the participants’ physical and mental health conditions, as well as some employment information, is given in addition. No significant differences were found between the analysis group (participants completed baseline and both 3- and 12-month follow-up) and those that dropped out after the baseline measurement. As for the analysis sample, there were only a few missing single items that were handled according to the particular instructions of the instrument’s manuals.

### 3.2. Knowledge and Attitudes Concerning Mental Health 

A variance analysis for repeated measures showed statistically significant differences in knowledge about mental illness over time, measured by MAKS (Table 2). Bonferroni-adjusted post-hoc testing displayed a significant improvement in between T1 and T3 (Table 3). There were no significant changes in social distance measured by SoDi. Table 2 represents the repeated measure ANOVA for the target variables over time in detail. Table 3 illustrates the Bonferroni-adjusted post-hoc testing for all significant changing scores.

**Figure 2:**
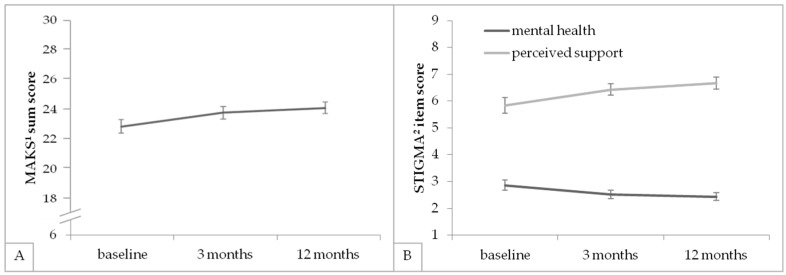
(**A**,**B**) Mean values and standard errors of the significantly changing scores at each measurement point. ^1^ Mental Health Knowledge Schedule [40]. ^2^ Stigma-related single items: Perception of own mental health condition and perceived support of colleagues with mental health problems at the workplace.

As for the six included stigma-related single items, a variance analysis for repeated measures with a Greenhouse–Geisser correction showed statistically significant differences in the perceived own mental health condition over time (Table 3). Bonferroni-adjusted post-hoc testing displayed a significant improvement in between T1 and T2, as well as in between T1 and T3. Also, a variance analysis for repeated measures showed statistically significant differences in the perceived support at the workplace over time (Table 3). Bonferroni-adjusted post-hoc testing displayed a significant improvement between T1 and T2, as well as between T1 and T3. No other significant changes could be found in the single items. Also, see Figure 2 for an illustration of the significantly changing scores.

### 3.3. Working Situation

No significant changes were found in the working situation during the observation period, as measured by the ERI and SI. See Table 2 for details.

## 4. Discussion

This investigation showed changes in stigma-related knowledge concerning mental health over a 1-year period after a specific manager training. Whereas for MAKS, significant improvements were found after 12 (though not after 3) months, no changes could be found comparing SoDi scores during the observation period. As literature reports both instruments to correlate, this discrepancy was unexpected [39]. Therefore, the nature of the changes needs to be discussed. First, a more content-related comparison of both instruments is necessary. By asking the participants about their willingness to get more closely in contact with a mentally ill person, SoDi focuses on attitudes by generating a personal involvement [49]. MAKS, on the other hand, uses items based on prejudices and thus focuses on knowledge-based attitude. Taking these differences into account, changes revealed in the data analyses might have been caused only by a change of the participant’s basic understanding and knowledge, and not by a substantial change in attitudes. In the case of such rather cognitive changes, it should be discussed whether the training could be improved by adding more emotionally focused parts that enhance the individual involvement.

However, another observation that needs to be discussed is the characteristic of the baseline SoDi score. Mean values appear to be very low on the scale at all three measurement points. In lack of a norm sample, the mean scores in this study were compared numerically to scores found in other investigations [40,50]. Thereby, it became apparent that participants of the present sample achieved considerably lower scores at baseline. Thus, they described their social distance to be very small and that they do feel very comfortable in the interaction with a person with a mental health problem. Thus, it could also be argued that floor effects covered any apparent changes. Several reasons might explain the very low baseline-scores. One consideration could be that the response patterns were traced by social desirability. This is plausible because the collaborating company has a very social working culture and is known for employee-oriented values. Managers that work within this environment might have internalized these values within loyalty and identification processes.

An even more comprehensible explanation for the very low scores is the sample composition. The large degree of voluntariness needed a great willingness of the participants to get in touch with the subject. That requires a fundamental interest in mental health topics concerning oneself or employee management. As described in the methods section, we also conducted a qualitative investigation by performing semi-structured interviews with some of the participants. Most of the interviewees presented themselves as very open-minded concerning mental health at the workplace. This qualitative insight into the manager sample might strengthen the idea of floor effects. The detailed results of the qualitative evaluation will be presented elsewhere.

Further on, single item analyses revealed a change in the participants’ perception of their own mental health condition. Thus, they described a better well-being at both follow-up measurements compared to the baseline measurement. Running preliminary analyses, we found significant improvements of objectively measured mental health (PHQ) after 3 months [1]. Within the analyses of the final data material, these improvements were not found anymore. Variations in the perception but not the objective mental health also indicate that participants did change in the way of thinking about mental health. This could be influenced by a better understanding, and thus evaluation of stress symptoms.

The training implemented within this investigation was based on an SMI program for managers that was conducted in another company from 2006 to 2008 [37,51]. A controlled long-term investigation of this previous intervention showed long-lasting effects over a period of 7 and 9 years [52,53]. Therefore, significantly better scores were found in the treatment group for participants’ mental health condition, as well as on the perceived working situation. As the training reported in the present article involves some basic parts of the stress intervention, we also wanted to see whether these effects could be reproduced within this shorter training version. As there were no changes in the scores, neither for work-related nor health-related instruments, efficacy cannot be shown in this data. The main difference between the two interventions was the focus on the managers’ employees. In the original training, participants were invited to think of any stressful situations at work and to identify individual resources. However, the training reported here mainly focused on stressful situations with employees and how to handle them within the team and the company.

We expected to find sustainable improvements in the managers’ knowledge and attitudes concerning mental health. Synoptically, not all expectations were met, as only one of two instruments revealed significant changes over the time. As discussed, this could be explained by several reasons and needs to be addressed in further investigations. First, the composition of the sample needs to be varied in order to avoid floor effects. For example, the training could be implemented at other locations in an obligatory way. Also, the proportion of managers with the highest school-leaving qualification in Germany was close to 90%. Investigation within production locations could bring more variety into this distribution. Above that, considerations regarding the difference between the two instruments should be addressed. There are other scales measuring attitudes and social distance concerning people with mental health problems [33,54,55] that should help considering a possible difference between attitudes in terms of social distance and attitudes based on (a lack of) pre-knowledge.

This pilot study showed first indications for the efficacy of a short intervention trying to support managers in their interaction with employees that suffer from a psychological illness. Based on the non-randomized and uncontrolled design, it is not possible to clearly ascribe these improvements to the training. Other variables, which we did not measure, could have influenced the findings. Due to the selective sample, generalization should also be made very carefully. Nevertheless, results indicate that a change in the participant’s attitudes somehow did take place within the observed period of time. Certainly, randomized controlled trials are needed in the future to learn more about the trainings efficacy in the working world.

### Limitations

A limiting factor of the study is the large amount of dropouts after the first measurement point. This might have been caused by our way of recruiting participants during the training and giving them time in site to fill in the questionnaires. When it came to the second and third measurement, response rate decreased. Another limiting factor is the unbalanced gender ratio of the sample. The very high proportion of male participants reflects the gender ratio of managers in the collaborating company. Still, the lack of female participants should be taken into account when interpreting the results.

## 5. Conclusions

This pilot investigation showed improvements in stigma-related knowledge concerning mental health over an observation period of 12 months after a single day of manager training. This indicates that even with short interventions, long-lasting benefits can be obtained. Interestingly, only one of two attitude-related instruments revealed significant effects. This brings up further considerations about the effect factors of the training and about how the training could be adjusted. Based on the uncontrolled design of the study, interpretations concerning the efficacy of the training need to be stated carefully. Yet, it can be argued that there has been some process within the observed group that might have been evoked by the intervention. Further research is needed to learn more about the concrete effects.

## Figures and Tables

**Table 1:** Demographic, employment and health information at baseline for all included participants and divided into response behavior.

	Baseline, 3- and 12-Month Follow-Up (*n* = 41)	Baseline and No Follow-Up (*n* = 26)	All Included Participants at Baseline (*n* = 67)
Male gender *n (%)*	35 (85.4)	21 (80.8)	56 (83.6)
Age at baseline *M (SD)*	47.8 (8.4)	45.6 (8.4)	47.0 (8.4)
Abitur or comparable *n (%)*	35 (87.5)	23 (88.5)	58 (89.3)
Position ^1^			
Stage A *n (%)*	4 (9.8)	1 (3.8)	5 (7.6)
Stage B *n (%)*	16 (39.0)	11 (42.3)	27 (40.9)
Stage C *n (%)*	8 (19.5)	7 (26.9)	15 (22.4)
Stage D *n (%)*	3 (7.3)	2 (7.7)	5 (7.5)
Others ^2^ *n (%)*	10 (24.4)	4 (15.4)	14 (20.9)
Personnel responsibility *M (SD)*	21.3 (36.1)	42.4 (98.3)	29.3 (67.2)
Working hours/week *M (SD)*	44.2 (8.6)	42.4 (9.8)	43.6 (9.0)
SF-12 ^3^ physical health	53.1 (6.8)	51.3 (6.4)	52.5 (6.7)
SF-12 ^3^ mental health	48.8 (9.0)	49.8 (9.6)	49.2 (9.2)
PHQ-D ^4^	4.1 (2.8)	3.8 (3.3)	4.0 (3.0)

^1^ Position in the company ranging from stage A (lowest) to stage D (highest). ^2^ Managers without a classifiable position, e.g., top management. ^3^ 12-Item Short Form Health Survey [43]. ^4^ Depression Scale of the Patient Health Questionnaire [44]. *M,* mean score. *SD,* standard deviation.

**Table 2:** Repeated measure ANOVA for the target variables over time.

*n* = 41	*M_T1_ (SD)*	*M_T2_ (SD)*	*M_T3_ (SD)*	*df*	*F*	*p*	η^2^_p_
SoDi ^1^	0.96 (0.63)	0.85 (0.66)	0.84 (0.65)	2, 82	1.84	0.165	0.04
MAKS ^2^	22.88 (2.89)	23.79 (2.75)	24.12 (2.53)	2, 82	5.77	0.005 *	0.12
STIGMA mh ^3^	2.86 (1.28)	2.52 (1.02)	2.43 (0.94)	1.52, 62.12 ^a^	5.61	0.011*	0.12
STIGMA shames ^4^	4.79 (2.41)	4.90 (2.10)	4.40 (1.98)	2, 82	2.14	0.124	0.05
STIGMA shameo ^5^	2.81 (1.63)	2.81 (1.71)	2.69 (1.66)	2, 82	0.22	0.801	0.01
STIGMA support ^6^	5.83 (1.91)	6.43 (1.43)	6.67 (1.43)	2, 82	4.42	0.012 *	0.10
STIGMA sd ^7^	2.40 (1.74)	2.57 (1.80)	2.45 (1.73)	2, 82	0.24	0.790	0.01
STIGMA inf^8^	4.40 (2.39)	4.60 (2.39)	4.86 (2.50)	2, 82	1.26	0.289	0.03
ERI ^9^ Effort	17.29 (4.09)	16.15 (4.89)	16.10 (4.66)	2, 80	2.83	0.065	0.07
ERI ^9^ Reward	47.88 (7.20)	48.66 (7.07)	48.07 (7.56)	2, 80	0.27	0.765	0.01
ERI ^9^ Overcommitment	2.37 (0.61)	2.32 (0.58)	2.31 (0.59)	2, 82	0.41	0.666	0.01
IS ^10^ general score	19.80 (9.20)	20.45 (9.54)	19.71 (9.88)	2, 82	0.35	0.705	0.01

^1^ Social Distance Scale [40]. ^2^ Mental Health Knowledge Schedule [39]. ^3^ Single item, own mental health condition. ^4^ Single item, shame for own illness. ^5^ Single item, shame for illness of a family member. ^6^ Single item, support at the workplace. ^7^ Single item, social distance. ^8^ Single item, willingness to communicate own illness at the workplace. ^9^ Effort–Reward Inventory [46]. ^10^ Irritation Scale [47]. * Significant at the 0.05 level. ^a^ Greenhouse–Geisser corrected. T1–T3 measurement points at baseline, 3 and 12 months after the training. *M,* mean score. *SD,* standard deviation. *Df,* degrees of freedom. *F,* F-value. η^2^_p_, partial eta square.

**Table 3:** Bonferroni-adjusted post-hoc comparison.

	Time	Time	Mean Difference	*SE*	*p*	Cohen’s *d*
MAKS ^1^	1	2	−0.90	0.38	0.062	−0.32
	2	3	−0.33	0.36	1.000	−0.13
	1	3	−1.24 *	0.40	0.010	−0.46
STIGMA mh ^2^	1	2	0.33 *	0.11	0.009	0.29
	2	3	0.10	0.12	1.000	0.09
	1	3	0.43 *	0.17	0.043	0.38
STIGMA support ^3^	1	2	−0.60 *	0.24	0.047	−0.36
	2	3	−0.24	0.27	1.000	−0.17
	1	3	−0.83 *	0.32	0.041	−0.50

^1^ Mental health knowledge schedule [39] ^2^ Single item, own mental health condition. ^3^ Single item, perceived support at the workplace. * Significant at the 0.05 level. *p, p*-value. *SE,* standard error.

## Appendix A. Stigma-Related Single Items (STIGMA)

**How do you think about yourself and your psychological condition?**	**label**
1. From a psychological point, I am entirely healthy.	mh
2. Would you feel embarrassed if being affected by a mental health problem (e.g., depressive disorder, anxiety disorder, alcohol addiction)?	shames
3. Would you feel embarrassed if somebody of your family would be affected by a mental health problem (e.g., depressive disorder, anxiety disorder, alcohol addiction)?	Shameo
**How are employees with mental health problems treated at your company?**	
4. Colleagues with mental health problems are supported by colleagues and supervisors and treated fairly.	support
5. Personally, I avoid dealing with colleagues with mental health problems, if possible.	sd
6. If I would have mental health problems, I would tell colleagues and supervisors at the workplace.	inf
